# Preserving Visual Functions During Gliomas Resection: Feasibility and Efficacy of a Novel Intraoperative Task for Awake Brain Surgery

**DOI:** 10.3389/fonc.2020.01485

**Published:** 2020-09-02

**Authors:** Marco Conti Nibali, Antonella Leonetti, Guglielmo Puglisi, Marco Rossi, Tommaso Sciortino, Lorenzo Gabriel Gay, Umberto Aldo Arcidiacono, Henrietta Howells, Luca Viganò, Paola Cosma Zito, Marco Riva, Lorenzo Bello

**Affiliations:** ^1^Neurosurgical Oncological Unit, Department of Oncology and Hemato-Oncology, Università degli Studi di Milano, Milano, Italy; ^2^Laboratory of Motor Control, Department of Medical Biotechnologies and Translational Medicine, Università degli Studi di Milano, Milano, Italy; ^3^Department of Anesthesia and Intensive Care, Humanitas Research Hospital, IRCCS, Rozzano, Italy

**Keywords:** brain mapping, optic radiation, visual outcome, gliomas, awake surgery

## Abstract

**Objective:** The intraoperative identification and preservation of optic radiations (OR) during tumor resection requires the patient to be awake. Different tasks are used. However, they do not grant the maintenance of foveal vision during all testing, limiting the ability to constantly monitor the peripheral vision and to inform about the portion of the peripheral field that is encountered. Although hemianopia can be prevented, quadrantanopia cannot be properly avoided. To overcome these limitations, we developed an intra-operative Visual field Task (iVT) to monitor the foveal vision, alerting about the likelihood of injuring the OR during task administration, and to inform about the portion of the peripheral field that is explored. Data on feasibility and efficacy in preventing visual field deficits are reported, comparing the outcome with the standard available task (Double-Picture-Naming-Task, DPNT).

**Methods:** Patients with a temporal and/or parietal lobe tumor in close morphological relationship with the OR, or where the resection can involve the OR at any extent, without pre-operative visual-field deficits (Humphrey) were enrolled. Fifty-four patients were submitted to iVT, 38 to DPNT during awake surgery with brain mapping neurophysiological techniques. Feasibility was assessed as ease of administration, training and mapping time, and ability to alert about the loss of foveal vision. Type and location of evoked interferences were registered. Functional outcome was evaluated by manual and Humphrey test; extent of resection was recorded. Tractography was performed in a sample of patients to compare patient anatomy with intraoperative stimulation site(s).

**Results:** The test was easy to administer and detected the loss of foveal vision in all cases. Stimulation induced visual-field interferences, detected in all patients, classified as detection or discrimination errors. Detection was mostly observed in temporal tumors, discrimination in temporo-parietal ones. Immediate visual disturbances in DPNT group were registered in 84 vs. 24% of iVT group. At 1-month Humphrey evaluation, 26% of iVT vs. 63% of DPNT had quadrantanopia (32% symptomatic); 10% of DPNT had hemianopia. EOR was similar. Detection errors were induced for stimulation of OR; discrimination also for other visual processing tract (ILF).

**Conclusion:** iVT was feasible and sensitive to preserve the functional integrity of the OR.

## Introduction

In contemporary neurosurgical oncology, the preservation of full functional integrity is crucial to grant patients' quality of Life (QoL) (1–3). During surgery, the use of brain mapping techniques allows for identification of neurological and cognitive functions (4–9). In this regard, preservation of the visual field is still challenging. Resection of intra-axial lesions involving the temporal, parietal, or occipital lobe may result in permanent visual field deficits such as *quadrantanopia* or *hemianopia* due to damage of the optic radiation (OR), the visual cortex, or both. Clinicians often underestimate the occurrence of visual field impairment. However, it is highly debilitating for the patient's daily living, particularly in cases of hemianopia that strongly impair exploration of the environment and performance of many everyday activities such as reading or driving. Quadrantanopia, instead, may be asymptomatic. These limitations result in a significant decrease in subject independence and work capacity and limit employment or leisure activities, increasing the risk of developing depression (10). Presently, the available intraoperative tools are not entirely accurate in detecting the OR and preserving visual field integrity in most clinical conditions. Attempts to use Visual Evoked Potentials during resection under general anesthesia failed to achieve consistent results (11–15). At the moment, the intraoperative identification of OR under awake conditions could thus be regarded as the optimum clinical tool. The standard is the Double Pictures Naming Task (DPNT), where the patient is asked to report the occurrence of any visual impairment during the presentation of two items located diagonally on a screen (16, 17). Although quite efficient in several conditions, this approach is limited in the detection of peripheral stimuli, as the size of the stimuli is large, and part of them may fall within the central portion of the screen. Moreover, the duration of the stimulus is long (the stimuli remain fixed on the screen until the next item is presented). These characteristics increase the likelihood that the subject uses alternative strategies such as foveal vision to detect stimuli. Other groups tried to overcome this limitation by using a virtual reality headset, which provides luminous stimuli in different parts of the screen (18). The duration of the stimuli in this study is short; however, the subjects were required to merely report whether they saw (detection) a luminous spot stimulus, but not to describe it (discrimination). To overcome these limitations, we developed a new Intraoperative Visual Task (iVT), designed to continuously monitor the detection but also the discrimination of the stimuli, requiring the patient to keep the foveal vision on a central fixation point while peripheral targets are shown peripherally. This enables the neuropsychologist to alert the surgeon as to when central fixation is lost during its administration, preventing the occurrence of false-negative responses.

We here present data on the intraoperative feasibility and efficacy of this new task: we retrospectively reviewed a series of patients with tumors involving OR who were submitted to resection under asleep-awake-asleep anesthesia where iVT was administered to detect and preserve OR. We compared their results with those of a previous homogenous cohort of patients where OR were mapped with the standard task (DPNT).

## Materials and Methods

### Patients

Patients consecutively admitted to the Unit from November 2014 to November 2019 were enrolled if they fulfilled the following inclusion criteria: (1) presence of an intra-axial lesion in close morphological relationship with the OR in at least in one segment (specifically tumors located in the temporal, or parietal or occipital lobe, or in the temporo-parietal junction [TPJ]); (2) the surgical approach or resection could affect the OR at any extent; (3) absence of any visual field deficits at pre-operative evaluation (assessed by Humphrey evaluation); (4) the patient was a candidate for resective surgery under asleep-awake-asleep anesthesia according to physical and cognitive performance and after a formal interview with a psychotherapist.

Patients were categorized in two groups:

***a) iVT Group:*** All patients operated on from November 2016 to November 2019 and where intraoperative Visual Test was used to map the OR;
***b) DPNT group***: All patients operated on from November 2014 to October 2016 before the introduction of the iVT, where the Double Pictures Naming Task was used to track OR.

Demographic, clinical, and imaging features were recorded (Table 1).

**Table 1:** Demographic and clinical variables of the patients belonging to iVT and DPNT groups.

**Variables**	**iVT Group**	**DPNT Group**	***P***
***N°* of patients**	54	38	
**Gender - N° (%)**			0.000^*^
Male	22 (40.7)	31 (81.6)	
Female	32 (59.3)	7 (18.4)	
**Mean age in years (**± **SD)**	41.6 ± 12.3	42 ± 12.3	0.85
**Mean years of Educational level (range)**	15.1 (8–17)	15 (8–17)	0.99
**Previous Treatments - N° (%)**			0.191
Yes	6 (11.1)	8 (21.4)	
No	48 (88.9)	30 (78.9)	
**Grade accord. WHO - N° (%)**			0.101
I	8 (14.8)	0 (0)	
II	22 (40.8)	18 (47.4)	
III	14 (25.9)	11 (28.9)	
IV	10 (18.5)	9 (23.7)	
**IDH-1/ IDH-2 mutated - N° (%)**			0.27
Mutated	30 (60.0)	27 (71.1)	
Wildtype	20 (40.0)	11 (28.9)	
**Hemisphere affected - N° (%)**			0.293
Right	21 (39.6)	11 (28.9)	
Left	32 (60.4)	27 (71.1)	
**Lobe affected - N° (%)**			0.650
Temporal	26 (48.1)	22 (57.9)	
Parietal	17 (31.5)	10 (26.3)	
TPJ	11 (20.4)	6 (15.8)	
**Lesion Volume, cm**^**3**^			0.124
Median	18.361	16.982	
Mean	29.564	30.278	
Range	0.7–144.52	4.87–99.763	
**Extent of Resection - N° (%)**			0.444
GTR (>/= 100)	44 (81.4)	29 (76.3)	
STR (99–90)	5 (9.3)	6 (15.8)	
PR(<90)	5 (9.3)	3 (7.9)	
**Initial Post-op DEX Dose (mg/kg)**			
	0.2	0.2	

Initial post-operative DEX dose refers to the dosage administered in the first three post-operative days; dosage was then tapered down within three weeks after surgery. iVT, intraoperative Visual Task; DPNT, double pictures naming task; N, number; SD, standard deviation; TPJ, Temporo-Parietal-Junction; DEX, Dexamethasone. Significant differences (p < 0.05) between the two groups are indicated by ^*^ and marked in bold.

All patients gave informed written consent to the procedure and the study was covered by IRB-1299.

### Neuroradiological Evaluation and Post-operative Imaging Processing

A pre-operative 3T MR (Philips-Intera) was used for tumor morphological and volumetric assessment. The protocol included: (1) axial 3D-FLAIR, (2) post-Gd-3D-T1-weighted fast-eld-echo, and (3) diffusion-weighted imaging and apparent diffusion coefficient diffusion-weighted imaging. Postoperative diffusion-weighted-MR was also performed to check for ischemic damage. Volumetric analysis was used to measure tumor volume. Volume was computed onto FLAIR volumetric sequences with semi-automatic segmentation using iPlan-Cranial-software (Brainlab, AG). A subgroup of 10 patients of iVT group underwent a High-Angular-Resolution-Diffusion-Imaging (HARDI) optimized diffusion sequence for clinical purposes using an 8-channel head coil. A spin echo, single shot EPI sequence was used with 73 directions collected with a b-value of 2,000s/mm^3^ and seven interleaved non-diffusion weighted (b0) volumes (TE:96ms, TR:10.4ms). The sequence had a matrix size of 128 × 128 with an isotropic voxel size of 2 mm^3^. Diffusion pre-processing was performed using Explore DTI, and HARDI spherical deconvolution whole brain deterministic tractography was modeled using StarTrack-software (19) (www.mr-startrack.com). The OR, inferior-longitudinal-fasciculus (ILF), inferior-fronto-occipital-fasciculus (IFOF), vertical-occipital-fasciculus (VOF), and arcuate-fasciculus (AF) were dissected in the ipsilesional hemisphere in all patients using TrackVis and the distance from the stimulation site was calculated in mm (20, 21). For each patient, the resection cavity identified on the 1 month postoperative T1-weighted image was registered to the preoperative diffusion imaging to identify extent of white matter resection (22). If over 50% of the streamlines were within the resection cavity, the tract was classed as resected (23).

### Surgical Procedure

Surgery was performed under asleep-awake-asleep anesthesia with total-intravenous-anesthesia (TIVA) with propofol and remifentanil and a laryngeal mask; curare was avoided. Motor, language, praxis, and cognitive mapping were performed according to the clinical context (4, 9, 23–26) with visual mapping. Surgery was aimed at obtaining a complete resection whenever feasible. A craniotomy was tailored to expose the tumor area and a limited amount of surrounding tissue. In the awake phase, we first tested and set the working current defined as the lowest current intensity inducing anarthria during counting or naming task when the probe was applied over the ventral Pre-Motor area. The same current intensity was then used throughout the entire cognitive and visual testing. Cortical mapping was used to identify the cortical safe entry zone, while subcortical mapping was initiated since the beginning at the tumor periphery to define the functional boundaries. In case of temporal tumors, language, and cognitive mapping was initially performed (naming and semantic association task). The visual mapping was started after the identification of the ILF and IFOF. In case of parietal or temporo-parietal junction tumors, language mapping was used to locate ILF and AF, Hand-Manipulation-Task (HMT) (9) to identify the parietal-frontal praxis network, followed by visual mapping. Once functional subcortical sites surrounding the tumor were all identified and the tumor functionally disconnected, resection of the mass was completed under general anesthesia, with the aid of motor mapping (when needed), MEP, and SEP monitoring. Histology was classified according to the last WHO Brain Tumor Classification (27).

### Intra-operative Neurophysiological Protocol

#### Brain Monitoring

EEG and Electrocorticography (ECoG) were continuously monitored to assess the depth of anesthesia and occurrence of subclinical seizures or after discharges. Free running EMG activity was monitored with a multichannel recording setup: up to 24 muscles were recorded from contralateral and ipsilateral muscles. The recording system monitored: (i) free-running background EMG activity; (ii) motor responses to brain mapping stimulation, (iii) MEPs evoked by stimulation of M1 with Train-of-Five High-Frequency (HF) technique throughout the procedure to monitor the integrity of descending motor pathways. MEPs were recorded from strip electrodes during the resection time, and from transcranial electrodes from incision to closure.

#### Brain Mapping

Low-Frequency (LF) stimulation or HF constant current stimulation was adopted for both cortical and subcortical mapping (4). LF was delivered by a bipolar probe, HF by a monopolar probe. Anodal polarity was used for HF cortical stimulation and cathodal polarity for subcortical stimulation. For cognitive mapping (language, visual) the HF repetition rate was increased to 3Hz (28). The lowest current intensity producing interferences when stimulating the ventral-premotor cortex (vPM), while the patient was performing naming or counting, was used throughout the mapping.

### Assessment of Neuropsychological Profile and Visual Field Abilities

An extensive neuropsychological evaluation was performed before, immediately (5–7th day after surgery), and 1 month after surgery by two board-certified neuropsychologists (A.L. & G.P.). The battery tests assessing language, memory, praxis and visuo-construct abilities, attention, and executive functions was used to depict the cognitive profile and reliability of responses to the visual assessment (23).

Visual field was evaluated by using the bimanual task (during the neurological exam) along with the Humphrey automated perimetry. Pre-operatively, the Humphrey test was performed within 15 days from admission by a board-certified ophthalmologist, along with bimanual task which was also repeated at admission. Only patients with no visual field deficits (at Humphrey evaluation) were enrolled in the study. During the post-operative period, each patient was evaluated every day during the first week by bimanual task, and data obtained at 7 days (T1) and 1 month after surgery (T2) were collected. Humphrey perimetry was repeated 1 month after surgery. Deficits on the Humphrey test were categorized as: (a) no deficit, when no alterations were detected; (b) quadrantanopia, when a defect (of any extent) was documented in the superior or inferior contralateral visual field; (c) hemianopia, when a defect was documented in the superior and inferior contralateral visual field. During all examinations, patients were also asked to report any subjective visual field disturbances interfering with normal day activities (reduction, blurring, or sparks in the contralateral visual field).

### Intraoperative Visual Tasks

**Figure 1:**
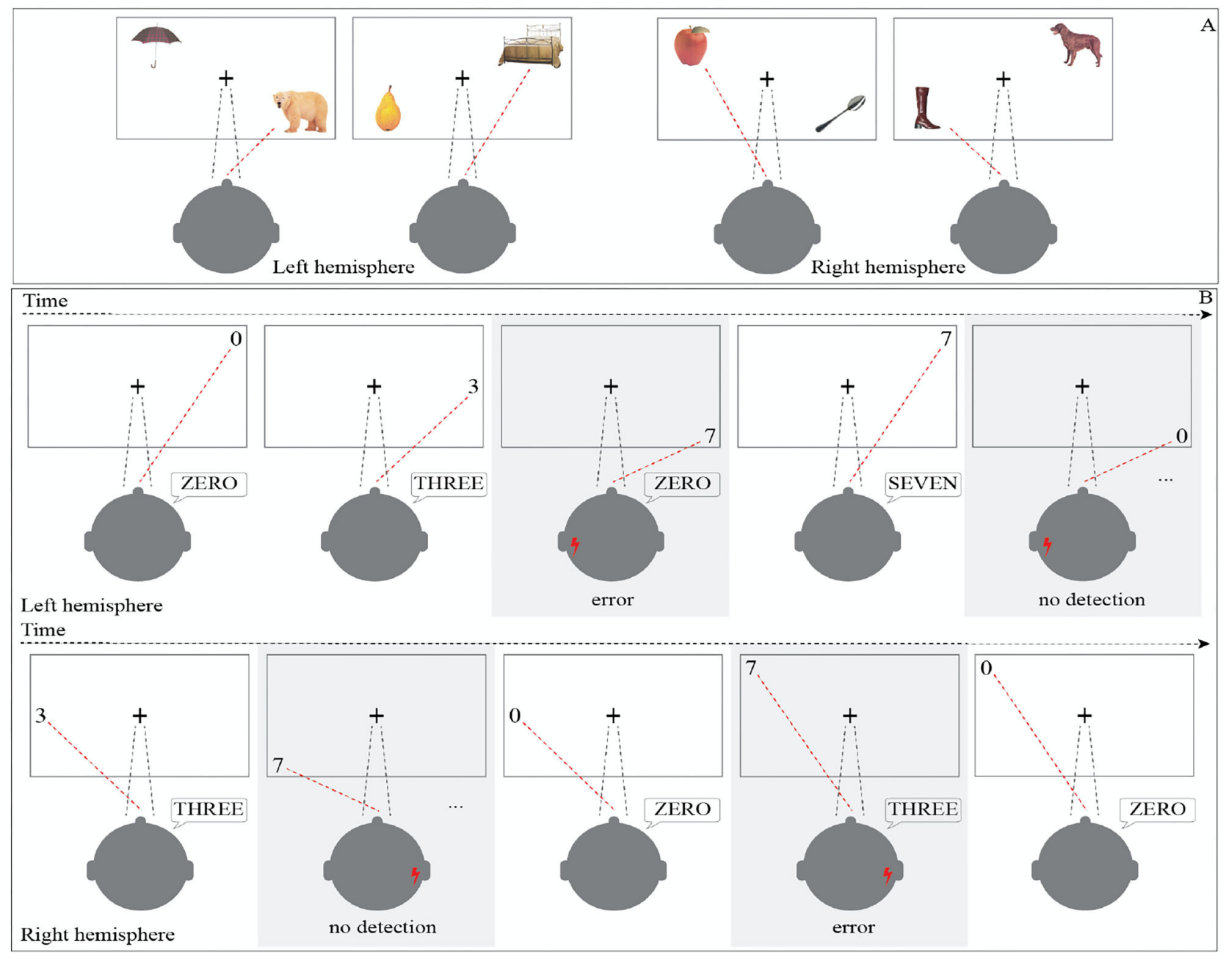
Intraoperative visual field tasks. **(A)** DPNT task: it consists in a modified version of the 4-screen naming task; two color items, selected out of Catricalà naming test items according to patient's performance, are placed diagonally (one in the quadrant planned to be tested and the other in the inferior contralateral side) on a 15” tablet screen. Patients during the test execution are invited to fix a red cross placed in the center of the screen, and to name both items. **(B)** iVT Intraoperative task: one number at a time was presented on a 15” screen and the patient was asked to name the number presented in the upper, lower, or middle (randomly) portion of the screen to allow the investigation of each quadrant of the peripheral visual field. In the upper and lower part of the figure the set up for the left or right hemisphere is, respectively, reported. The answer of the patient is reported in the bubble. The gray square and red lightning bolt represent the time at which the DES was applied over the investigated site.

Two tasks were used. The Double Pictures Naming Test (Figure 1A), as previously described (17), consists in a modified version of the 4-screen naming task; two color images were selected according to the patient's preoperative performance from a naming test adapted for Italian Population (29). Images are placed diagonally (one in the superior of inferior quadrant planned to be tested and the other in the inferior or superior contralateral side) on a 15” tablet screen. Patients during the test execution are invited to fix a red cross placed in the center of the screen, and to name both items.

The iVT consisted in the presentation of three different target numbers−0, 3, or 7—appearing randomly, one at a time, for 0.3 to 0.5 s (according to pre-operative patient performance), on the left or right extreme half of a 15” tablet screen, either at the upper, middle, or lower portion. The subject was instructed to continuously fix a cross positioned in the center of the screen, matching with his/her foveal vision. The patient was asked to name the series of target numbers falling in the extreme portion of his/her peripheral visual field (Figure 1B). The use of different numbers allowed the neuropsychologist to assess that, during each trial, the patient's response was consistent, avoiding false positive or negative responses. The numbers 3, 0, or 7 were chosen because they are different in shape, to avoid errors based on the recognition of numbers of similar shape (i.e., 1 and 7; 3 and 8; 0 and 9). Maintenance of the gaze on the fixation point was assessed by the neuropsychologist, who directly monitored the patient's eyes and reminded the patient to continuously fix the cross in the center of the screen during task execution, before the appearance of each item. The same neuropsychologist who trained the patient in the pre-operative stage deployed the test intraoperatively.

During the procedure, when a stimulation interfered with the task, an interval of 3–4 s preceded the next stimulation to allow the patient to regain stable task performance. A stimulation site was deemed effective when it interfered with the task for at least three non-consecutive trials. During DPNT execution, the interferences consist in naming errors. During iVT performance, the type of interference (errors) was categorized as a “detection error” (clear loss of vision; the patient was not able to see the number, in one specific region—inferior, central, superior—of the peripheral field) or as a “discrimination error” (hesitation in reporting the number or in mistake in number recognition).

Patients with temporal lesions were positioned supine, with a 30° elevation of the head that was slightly tilted toward the contralateral side of the tumor. Patients with parietal tumors were positioned laterally, lying on the side contralateral to the lesion. In both surgical positions, both eyes were free to watch the screen.

### Feasibility of iVT and Functional Efficacy of Visual Examination Tasks

Feasibility of the iVT was assessed by looking at ease of administration (pre-operatively and intraoperatively) and the number of patients who successfully completed the iVT during surgery. We evaluated the ability of the neuropsychologist to assure the maintenance of central fixation point during task execution and to detect any interference in peripheral vision during task performance due to stimulation.

Functional efficacy of iVT and DPNT was evaluated by visual field analysis, performed either by the bimanual task during neurological examination or Humphrey perimetry 1 month after surgery. In addition, patients were asked to refer any subjective visual field disturbances, possibly interfering with normal life activities.

### Impact on EoR

We measured the Extent of Resection (EOR) using a 1-month post-operative MR (volumetric FLAIR for non-enhancing lesions—target of resection-, and post-Gd T1-weighted images for enhancing lesions—target of resection). The FLAIR hyperintense or T1-weighted Gd-enhanced signal abnormalities were included in the lesion load for non-contrast enhancing lesion or high-grade gliomas, respectively, and were reported in cubic centimeters. The EOR corresponds to the percentage of the volume resected with respect to the preoperative volume: (preoperative volume–postoperative volume)/preoperative volume and were classified as follow: Gross Total Resection, (GTR) EOR = 100%; subtotal resection, 100 < EOR < 90%; Partial < 90%. For the analysis, Subtotal and Partial resection were merged (30).

### Intraoperative Recordings of Stimulated Sites During iVT

**Figure 2:**
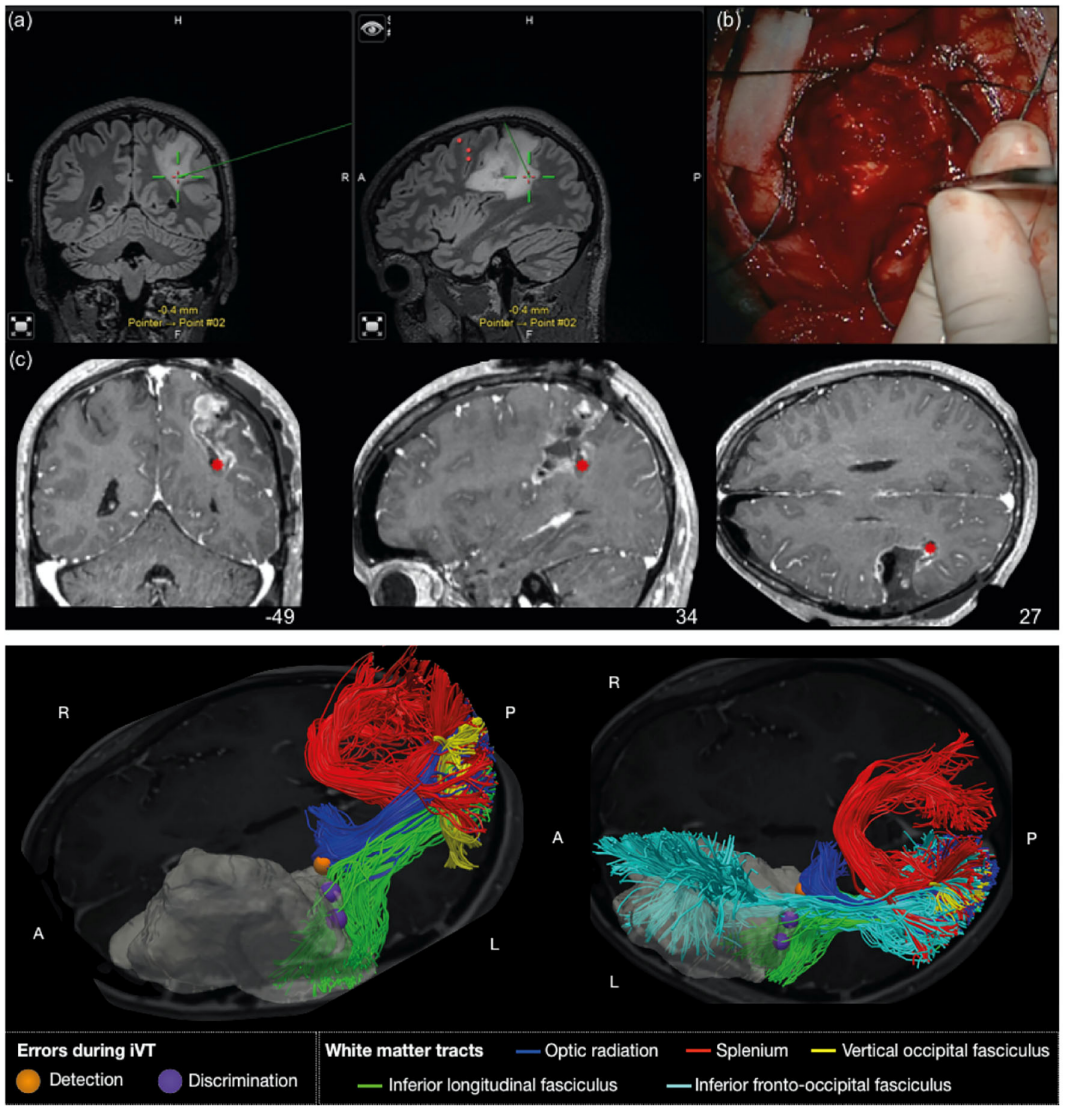
Representative examples of glioma cases in which the iVT was performed intraoperatively for OR detection and preservation. A case of presumptive right parietal lower-grade glioma. This young male patient had a recent clinical history of sensory-motor seizures. A right tumor mass involving the parietal lobe was documented at the MR study; representative **(a)** coronal—left panel and sagittal—right panel. FLAIR Images, taken as a snapshot during surgery (the red dots indicate the cortical sites corresponding to M1). The patient was operated on in asleep-awake-asleep anesthesia, and submitted during the awake phase to subcortical language, haptic, cognitive, motor, and visual (iVT) mapping. While the patient was performing iVT several sites interfering with the task execution during subcortical DES (LF, 4 mA) were identified. A site where DES induced a detection error is shown **(b)** in the intraoperative picture on the operative field and tracked with the neuronavigation probe. The location is indicated in the intraoperative snap shots **(a)** as a green arrow. The location of this site was also reported, co-registered, and superimposed on the post-operative postGD-T1 weighted MR **(c)**, coronal, sagittal and axial images, as a red dot. Histo-molecular diagnosis documented a grade III IDH1 mutated, MGMT methylated astrocytoma. A case of right fronto-temporo-insular presumptive lower-grade glioma, in a 35-year-old male patient. The patient was operated on in asleep-awake-asleep anesthesia, and submitted to motor, cognitive, haptic, motor, and visual (iVT) mapping. The tumor volume is represented in gray in the brain3D reconstruction along with reconstructed tracts (HARDI) surrounding and/or infiltrating the tumor mass: on the left is the axial view from above; on the right is the axial view from below; P, posterior; A, anterior; L, left; R, right. The sites giving interferences while stimulated during iVT task (LF 3 mA) are superimposed to the map as dots. Detection errors are in orange, while discrimination is in purple. Detection errors correspond to OR; discrimination to ILF. The names of the reconstructed tracts are reported in the legend.

The 3D DICOM coordinates of the sites giving responses at DES during iVT performance were recorded by Neuronavigation (Curve, Brainlab, AG), at the end of the subcortical mapping procedure, just before the resection of the tumor (Figure 2). The recorded positive sites were verified offline on the video recordings, registered to the T1 and normalized to MNI (Montreal Neurological Institute) space using an affine transformation implemented in SPM8 software (23). The stimulation sites were entered into a white matter disconnectome tool (31) to identify likely stimulated tracts. Tracts were reported as stimulated when the probability of stimulation was over 50% in over 50% of patients.

### Statistical Analysis

Multiple chi-square tests were performed to assess for the influence of tumor location, affected hemisphere, tumor volume, gender, and histomolecular diagnosis on visual outcome and to compare results obtained with iVT or DPNT. Analysis were performed with IBM SPSS 22nd Version.

## Results

### Patients

Demographic, clinical, imaging, and histo-molecular data are reported in Table 1. The iVT group includes 54 patients; the DPNT group includes 38 patients. In both groups, tumors were located in the temporal lobe or at the temporo-parietal junction, of both hemispheres. Groups were homogeneous for all clinical and imaging variables but gender.

### Feasibility and iVT Interferences During DES

In the pre-operative stage, all patients were able to perform the iVT properly after the initial training instruction. Training time took 12 min on average. The pre-operative administration was used to assess the baseline performance and to tailor the interval time of item presentation (0.3 or 0.5 ms) to individual patient profile. In the theater, iVT was installed on the same tablet used for all task presentations for mapping. The iVT could be administered in both positions (i.e., supine or lateral) and all patients were able to complete the task. During task execution, the neuropsychologist was always able to assure the maintenance of the fixation point (either by looking at patient eyes and reminding the patient to fix the cross located in the central portion of the computer screen, generally every two or three items).

During stimulation, at least one visual field interference was recorded in all patients, consistent with reaching the OR. Interference was clear in the case of temporal tumors, consisting of a clear loss of either the inferior, central, or superior peripheral vision (the patient was not able to specifically see the number in one of the portions of the peripheral field: i.e., Detection Error) (Table 2). In the case of parietal tumors, more careful administration was required: interferences consisted either of Detection errors in the inferior, central, or superior field, or in hesitation or mistakes when reporting the number (Discrimination Error) (Figure 2 and Table 2).

**Table 2:** Classification and number of interferences evoked by Direct Electrical Stimulation during patient intraoperative Visual Test performance according to the different tumor location.

**Location**	**N° of cases**	**Detection errors**	**Discrimination errors**	***p***
Temporal	26	64 (71.91%)	25 (28.1%)	0.001^*^
Parietal	17	13 (22.41%)	45 (77.59%)	0.001^*^
TPJ	11	16 (55.2%)	13 (44.8%)	0.001^*^

“Detection errors” were more frequently induced during resection of temporal tumors and they consisted in clear loss of either the inferior, central, or superior peripheral visual field (the patient was not able to specifically see the number in one of the portion of the peripheral field); “Discrimination errors” were mostly evoked during resection of parietal tumors and consisted in hesitation in reporting the number or in mistaken number recognition. Significant differences (p < 0.05) are indicated by ^*^ and marked in bold. TPJ: Temporo-Parietal-Junction.

The time requested for task administration, during the awake phase of the surgery, was, on average, 5 min for temporal or temporo-parietal junction tumors, and 7 min for parietal tumors.

Sites detected during iVT performance were also checked for naming and reading errors, and no overlap was observed. In addition, no sites were located close to those giving interferences during the HMT task.

### iVT Group—Functional Outcome

In the immediate postoperative period, 13 patients (24%) experienced visual disturbances, referring to either reduction (6 patients) or blurring or sparks in the contralateral visual field (all patients). One month after surgery, 50 (92%) patients did not report any subjective visual field disturbances and were asymptomatic. The Humphrey perimetry assessment showed that 36 patients (67%) did not have any visual field defect, 18 patients (33%) had an asymptomatic quadrantanopia, involving at a variable extent the superior of inferior contralateral visual field; no hemianopia was documented (Table 3).

**Table 3:** Permanent (1-month post-op) Visual Outcome of intraoperative Visual Test (iVT) and Double Pictures Naming Test (DPNT) groups as determined by Humphrey evaluation.

	**No deficit**	**Quadrantanopia**	**Symptomatic quadrantanopia^#^**	**Hemianopia**	
Group					0.001^*^
iVT (54)	36 (67%)	18 (33%)	0	0	
DNPT (38)	10 (26%)	24 (63%)	12 (32%)	4 (10%)	

*The total number of patients for each group is reported below the group name*.

# Indicates the patients who referred any subjective persistent contralateral visual field disturbances during post-operative examination at 1 month. Significant differences (p < 0.05) are indicated by ^*^ and marked in bold.

Visual outcome was not influenced by many clinical variables such as gender or histo-molecular diagnosis (Table 1). Conversely, it was affected by tumor side, location, and EOR. The rate of visual deficits was higher in the case of temporo-parietal junction tumors and in tumors in which a subtotal resection was achieved (Table 4).

**Table 4:** Association between clinical variables and visual outcome (assessed using Humphrey test) in the intraoperative Visual Test group.

	**No deficit**	**Quadrantanopia**	***P***
**Gender**			
M (22)	13/22 (59.1%)	9/22 (40.9%)	0.327
F (32)	23/32 (71.9%)	9/32 (28.1%)	
**Histo-molecular diagnosis**			
LGG (38)	26/38 (68.4%)	12/38 (31.6%)	0.673
HGG (16)	10/16 (62.5%)	6/16 (37.5%)	
**Side**			
Right (21)	11/21(52.4%)	10/21 (47.6%)	0.076
Left (33)	25/33 (75.8%)	8/33 (24.2%)	
**Location**			
Temporal (26)	19/26 (73.1%)	7/26 (26.9%)	0.007^*^
Parietal (17)	14/17 (82.4%)	3/17 (17.6%)	
TPJ (11)	3/11 (27.3%)	8/11 (72.7%)	
**EOR**			
>/=100% (45)	34/45 (75.6%)	11/45 (24.4%)	0.002^*^
<100% (9)	2/9 (22.2%)	7/9 (77.8%)	

Number of cases for each variables and the corresponding percentage are reported. Significant differences (p < 0.05) are indicated by ^*^ in bold.

### Extent of Resection

Complete resection was reached in 81.4% of patients of iVT group and 76.3% of cases of DPNT, respectively (Table 1). The achievement of subtotal or partial resection was due to the identification of subcortical sites interfering with language (4 cases), praxis functions (2 cases), or with the OR (4 cases) where tumor removal was deliberately stopped.

### iVT and DPNT Comparison

DPNT training time was 11 min on average, and intraoperative visual mapping time was 6 min on average, showing no difference with iVT. Conversely, the rate of immediate and permanent deficits was higher in the DPNT group (Table 3). Immediate visual disturbances in the DPNT group were registered in 84% vs. 24% of patients of the iVT. At one month, 16 patients (42.2%) in the DPNT group referred visual disturbances. Four patients were diagnosed with hemianopia, and 22 with quadrantanopia (12 of them referring some type of symptomatic visual disturbances interfering with normal life). On the contrary, EOR did not differ between the two groups (Table 1).

### White Matter Stimulation

**Figure 3:**
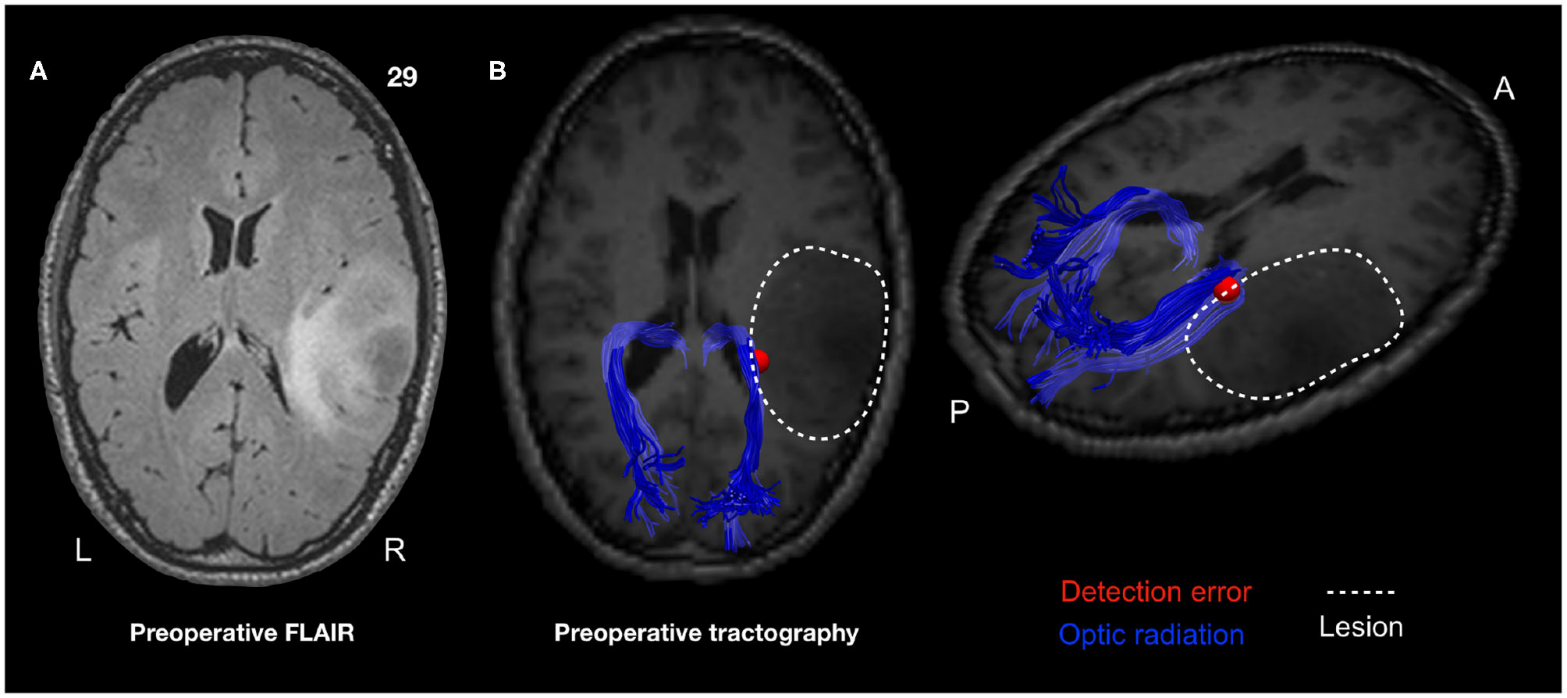
A case of presumptive right fronto-parietal lower-grade glioma in a 32-year-old female, operated on in asleep-awake-asleep anesthesia with the aid of motor, cognitive, haptic, language and visual (iVT) mapping. The axial FLAIR is in the upper left panel **(A)**. DES (LF 3 mA) interfered in multiple sites during iVT performance. The location of one of these (giving detection errors) is reported as a red dot in the pre-operative postGd T1 weighted MR to which the reconstructed OR (in blue, HARDI) are coregistered and superimposed **(B)**. The location of the site is within the lateral border of OR, in an area of tumor infiltration; the tumor volume is indicated with a dotted line (middle and right upper panels show the axial and the lateral view, A, anterior; P, posterior; L, left; R, right). Resection was stopped at this level, resulting in a subtotal resection. The histo-molecular diagnosis revealed grade II IDH1 wt astrocytoma.

Tractography was performed in 10 patients of the iVT group to compare patient anatomy with their intraoperative stimulation site (Figure 2 and Table 5). Five tracts of interest (OR, IFOF, ILF, splenium, VOF) were virtually dissected in all patients in the ipsilesional hemisphere and compared with the type of intraoperative errors and the impact of resection. The two tracts that were most commonly associated with errors were the OR and the ILF. Stimulation of the OR was associated with detection errors, while discrimination errors were more associated with the ILF. Interestingly, looking at the resection cavity, part of the OR and ILF could be resected, without determining permanent visual or reading errors detected during the visual or language mapping. In four patients (for example see Figure 3), the OR were severely infiltrated by the tumor; in these cases, detection errors were induced during surgery by stimulation of subcortical sites corresponding to OR, where resection was stopped.

**Table 5:** Clinical and imaging features, intraoperative findings, stimulated and resected tracts, and permanent visual outcome of 10 patients of iVT group in whom HARDI tractography was performed to visualize the relation of the site(s) of stimulation and the location of stimulated tracts, along with the resected tracts.

**Patient**	**Lesion location and hemisphere**	**Intraoperative error**	**Stimulated tracts**	**Resected tracts**	**Postoperative outcome**
1	Right fronto-temporo-insular	Discrimination and detection	ILF, OR	ILF (partial), OR (partial)	Quadrantanopia
2	Left occipital	Discrimination and detection	ILF, OR, VOF, splenium	ILF (partial), VOF	No visual
3	Right insula	Discrimination and detection	ILF, OR	ILF (partial)	No visual
4	Right temporal	Discrimination and detection	ILF, OR	ILF (partial)	No visual
5	Left posterior temporal	Discrimination and detection	ILF, OR	ILF (partial)	No visual
6	Left anterior temporal	Detection	ILF & OR	ILF (partial)	No visual
7 (Figure 3)	Right parietal	Discrimination and detection	ILF, OR	ILF (partial), OR (partial)	Quadrantanopia
8	Right anterior temporal	Discrimination	ILF, OR	ILF (partial), OR (partial)	Quadrantanopia (superior)
9	Left insula	Detection	ILF & OR	ILF (partial)	No visual
10	Right fronto-temporo-insular	Discrimination and detection	ILF & OR	ILF (partial)	No visual

## Discussion

The intraoperative identification and preservation of visual pathways requires the patient to be awake and fully cooperative. Different tasks have been proposed. Although efficient across most conditions, the main constraint of these approaches is that they do not completely grant the maintenance of foveal vision during all testing, limiting the ability to continuously monitor the patient's peripheral vision. These tasks avoid hemianopia but fail to prevent quadrantanopia in most cases. The ideal task for assessing visual pathways in the theater should be easy to use, able to assess both central and peripheral vision simultaneously and to inform about which portion of the peripheral field is encountered, and it should also give continuously insight on the maintenance of the central fixation point by the patient. The risk of false negatives due to eye movement is reduced since the patient must name the stimulus, thus confirming its detection. In addition, by considering that the OR, particularly in the posterior region of the temporal lobe or in the parietal lobe, are close to other tracts (such as the ILF or IFOF), the task should be able to potentially differentiate between responses evoked by the stimulation of the OR compared with neighboring tracts. The iVT was designed to fulfill these criteria, and in this work, we evaluated its performance in a series of patients with lesions involving OR, belonging to various clinical contexts.

Feasibility of the iVT was high: all patients regardless of education, gender, age, neurological conditions, and of glioma grading were able to learn, practice, and perform the task intraoperatively without limitations. It could also be used in the operative position (lateral or supine position). It did not require any change in the intra-operative armamentarium, as it was deployed through the same computer screen currently in use for other tasks by the attending neuropsychologist. The task was highly reliable, producing visual interferences in all patients. The risk of false negatives was decreased as each stimulus appeared on the extreme peripheral portion of the screen for a very limited time (0.3–0.5 s), improving the sensitivity of stimulus detection. False negatives were also reduced by the continuous monitoring of the patient's foveal vision during task performance by the neuropsychologist, who was continuously reminding the patient to look at the cross appearing at the center of the screen. The task contains three different numbers (3,0,7) that appeared on the extreme peripheral portion of the screen. These numbers were chosen because they were different in shape, avoiding errors based on the recognition of numbers of similar shape. The use of numbers, instead of picture naming, allowed us to avoid the occurrence of errors due to language interferences, because the ability to name a number is a strongly overlearned automatism, less impaired by language difficulties (32, 33). Furthermore, the iVT assesses the superior, medial, or inferior regions of the extreme peripheral field separately. The evoked interferences could be categorized as either detection errors with clear loss of peripheral vision or discrimination errors, when the patient hesitates or names the number incorrectly. The interferences were specific to saying the number, because in the same sites we did not observe any mistakes while the patient was performing language mapping (using a reading or picture naming task).

The type of errors occurred in different anatomical locations: detection errors were most frequently observed during temporal tumor resections, whereas for parietal tumors, stimulation initially induced discrimination errors, then detection errors when progressing medially with the resection. These findings are not surprising: to induce a clear loss of peripheral vision, stimulation should encounter compacted OR fibers, such as in the mesial temporal lobe, or inferior mesial parietal lobe; hesitation may result from the stimulation of a limited number or dispersed OR fibers, such as in the temporo-parietal junction, or to the stimulation of ILF. Interestingly, the ILF crosses the OR in the posterior part of the temporal lobe, and in the lateral temporo-parietal junction, where discrimination (i.e., hesitation or error in number recognition) errors are induced by DES while patient was performing the IVT. According to this view, the iVT may provide the surgeon with two types of information: discrimination errors may be encountered when stimulating the ILF or dispersed OR fibers, whereas detection errors may be encountered when stimulating the compacted OR, such as in the mesial temporal lobe or inferior and mesial parietal lobe. We also showed that, while both detection and discrimination errors could be induced during the iVT, detection errors were crucial in discriminating the OR from surrounding white matter tracts involved in other aspects of visual processing such as ILF, as evidenced with tractograghy. The ILF transfers information from visual regions to limbic and memory centers, and damage to the ILF results in visual agnosia or recent memory deficits. Given that resection of the ILF does not cause visual field deficits, it is crucial that visual errors during intraoperative mapping are specific to OR to avoid false positives. As the iVT is able to reveal different types of visual error in contrast with previously described approaches, the approach employed by the iVT greatly improves specificity of intraoperative mapping for OR, providing better information for the neurosurgeon about the underlying white matter and also about the risk of deficits. Analysis of resection cavities confirms these findings; in addition, it shows that at least a portion of the OR as depicted by tractography can be safely removed when iVT is used, calling for caution in using exclusively tractography reconstructions of the OR to guide tumor removal.

The high efficacy of the iVT was demonstrated as over 90% of patients experienced no visual disturbances in the post-operative period. Using Humphrey perimetry (1 month after surgery), nearly 70% of patients were completely normal following surgery. The comparison with the functional results obtained with DPNT further support this conclusion: iVT completely avoided hemianopia, while significantly reduced the occurrence of quadrantanopia, and specifically the number of patients who referred any type of post-operative subjective contralateral visual field disturbances possibly interfering with a normal life. In the iVT group no patients were diagnosed with a complete quadrantanopia vs. 32% of those in the DPNT group. The rate of deficits assessed using the Humphrey test was higher in non-dominant or in TPJ tumors; the presence of language tracts in the dominant side may have protected part of OR, and this may be missed in the non-dominant side. Tracking the OR in the TPJ seemed at least at the beginning to be more difficult, due to the coexistence of discrimination and detection errors (Table 2). The use of the iVT did not reduce the chance of achieving a complete resection, which globally was higher than 80%, without difference with the DPNT group. When a subtotal resection was only reached, the tumor removal was stopped due to functional reasons: language tracts or OR were severely or partially infiltrated by the tumor, and this may also explain the higher rate of visual deficits measured in these cases.

The main limitation of this study is the retrospective design. A selection bias of a surgical series, the progressive expertise and surgical confidence with the test over the study time are also to be acknowledged as further limitations. A larger prospective cohort will be needed to confirm these data.

## Conclusions

These results indicate that the iVT is a feasible and effective task to prevent the occurrence of visual deficits in the majority of patients with a tumor involving the OR, independent of age, sex, or tumor location.

## Data Availability Statement

The raw data supporting the conclusions of this article will be made available by the authors, without undue reservation.

## Ethics Statement

The studies involving HUMAN PARTICIPANTS was reviewed and approved by Humanitas Research Hospital Ethical Committee (IRB-1299).

## Author Contributions

MC, AL, GP, and LB: contributed conception and design of the study. LB: selected patients, directed, and executed the surgical procedure and the intraoperative brain mapping. MC, AL, GP, and HH: acquired data and performed statistical analyses. AL, GP, HH, LV, and MRo: produced figures. AL, MC, and LB: wrote the first version of the draft. MC, AL, GP, LV, MRi, HH, LG, MRo, UA, TS, and PZ: discussed and interpreted the results. All authors contributed to manuscript revision, read, and approved the submitted version.

## Conflict of Interest

The authors declare that the research was conducted in the absence of any commercial or financial relationships that could be construed as a potential conflict of interest.
